# The Relationship between Motor Function and Behavioral Function in Infants with Low Birth Weight

**Published:** 2016

**Authors:** Malek AMINI, Faranak ALIABADI, Mehdi ALIZADE, Majid KALANI, Mostafa QORBANI

**Affiliations:** 1 1.Department of Occupational Therapy, School of Rehabilitation sciences,Iran University of Medical Sciences, Tehran, Iran; 2 Pediatric Department, Iran University of Medical Sciences, Tehran, Iran; 3 Department of Public Health, Alborz University of Medical Sciences, Karaj, Iran

**Keywords:** Motor functions, Behavioral performance, Low birth weight infants

## Abstract

**Objective:**

Nowadays, the evaluation of all aspects of infant development is important. However, in practice, some of these assessments, especially those requiring more manipulation on high-risk infants, may impose additional stress on them.

Therefore, sometimes it is essential to utilize the results of a developmental assessment for the prediction of some other aspects of development. This study evaluated the relationship between the scores of the behavioral tests and the motor function test.

**Materials & Methods:**

This cross-sectional study and was undertaken in the Neonatal Intensive Care Center and Clinic of Shahid Akbar Abadi Hospital, Tehran, Iran. A group of 50 infants with low birth weights was selected based on the easy non-contingency method and the inclusion criteria, and served as the participants. In order to assess the motor function and the behavioral performance, the motor function test (a test of infant motor performance (TIMP)) and the neonatal behavioral assessment scale (neonatal behavioral assessment scale (NBAS)) were used respectively. TIMP has both stimulation and observation sections. The items include habituation, social interaction, motor system, state organization, state regulation, autonomic system, smile, supplementary items, and the reflex.

**Results:**

No significant association was found between the items of the habituation of behavioral testing and the observation of the movement test. There was no statistically significant relationship between the habituation and stimulation sections as well as between the system autonomous of the behavioral test and the observation section of the motor test (P>0.05). The relationship between other variables was statistically significant (P<0.05).

**Conclusion:**

The scores of some behavioral performance items could be a good predictor of the scores of the motor function items for low birth weight infants in the neonatal period.

## Introduction

Motor learning is one of the primary sources of learning. It provides great opportunities for the appropriate growth of motor and cognitive systems, and it makes strong connection between them (1). Because the motor skills have a major role in human life, measurement of motor skills has come into numerous researchers’ attention. Generally, one out of 10 children suffers from physical or mental impairments or disabilities, estimated to be 140 million children globally (2).

Risky children are more at risk of such problems than healthy children (3). Ten to fifteen percent of children that suffer from a major motor or behavioral disability have a history of hospitalization in NICU (2). Low birth weight (LBW) is considered as a risk factor in the child’s healthy conditions and communication (2, 4-7). High-risk infants of developmental disorders are one of the major challenges to their families, especially young parents who are not familiar with the motor developmental stages of infants, which cause the delays in development, are not considered. Hence, meticulous examination of the motor and behavior conditions via an accurate measure and, if necessary, the provision of early intervention for infants at risk can reduce the risk of developmental disorders in such newborns (8). 

The developmental assessment of infants can be carried out through various tests. The purpose of each of these tests is the evaluation of a specific aspect of development. 

For example, some are designed for motor assessment; the others mostly utilized for the neurological-behavioral assessment. These tests can be categorized based on other aspects, for instance, predictive, evaluative and discriminative tests (9).

One of the assessments that occupational therapists and physiotherapists persist on is the assessment of motor performance. Usually in the process of motor assessments, the infant is subject to the assessor manipulation, so it leads to stress in infants. If the situation of infants becomes more fragile, the stress levels increase (10). Therefore, carrying out some tests with greater manipulations in high-risk infants (i.e., with more fragile situation than healthy infants) is prohibited. 

This prohibition leads to some contradictions and paradoxes. Developmental Evaluation, including the motor evaluation, is more essential in high-risk infants than in healthy infants; nonetheless, there are some certain limitations on performing numerous tests on them. Another point is the variety of evaluations. A comprehensive assessment of the evaluation requires the implementation of a variety of assessments, which leads to the increase in the stress level of high-risk infants owing to the greater exposure to manipulations. Furthermore, it can be more costly, more time-consuming, and multiple assessments may not be accessible. That is why one of the major concerns of the evaluations cholars is to utilize the assessments, which provide the most amount of information about the infants development in the least amount of time and with the minimal manipulation. 

A good solution to this problem is answering to the question whether assessment of developmental aspects, which requires less manipulation (such as the neurobehavioral development aspects) on the aspects of assessment of developmental aspects which require more manipulation (such as the motor development aspects), provides useful and enough information or not? Finding the answer of this question can be regarded as major steps in the developmental assessment of risky infants, early diagnosis and early interventions, which leads to achievement of best results in development. A majority of the previous studies have measured the motor functions in infancy, and the behavioral and cognitive performance in the following years in their school age or adolescence. For example, Yvonne et al. and Falk et al. observed a statistically significant relationship between the motor function in the infants with LBW and its behavioral performance in the first year, and noted that both the motor function and behavioral performance of such infants are lower than the motor and behavioral performance of children with normal birth weight (11, 12). Consequently, infancy is the most sensitive age for getting stress via manipulation, and this stress will be exposed in their behavior in older ages. Many of studies have reported low motor and behavioral performance in infants with low birth weight; however, none of them have examined the possible interaction of them, particularly in infancy (9-11, 13, 14) Ohgi et al. designed a study in order to zero in on the efficiency of NBAS test in anticipating the future developmental disabilities and concluded that NBAS served as a predicator of future developmental problems (15). However, they did not investigate any probable relationship between motor and behavioral performance in the neonatal period. 

The present study was aimed at assessing the relationship between motor function and behavioral function in Infants with LBW. 

## Materials & Methods

In this section, the correlations between different items of the motor and behavioral functions of infants were estimated, and the scores of the motor function test were predicted through the scores of behavioral test of infants with LBW. In this study, conducted in the NICU and Infant Clinic of Akbar Abadi Hospital, Tehran, Iran, 50 infants up to 2 months of age, with LBW were selected using inclusion criteria and non-contingence sampling method.

The inclusion criteria were as follows: The corrected age of less than 2 months and more than 36 gestational weeks, birth weights between1500 and 2499g, the infant parental consent, the absence of co-existing disabilities, (including meningitis, encephalitis), asphyxia, hypoglycemia, sepsis, bleeding, surgery, hydrocephalus, microcephaly, seizures, feeding problems, having more than 5 apnea, anomalies in organs, and orthopedic abnormalities, such as hip dislocation and normal cranial ultrasound, all of which had been checked using the newborn records.

Exclusion criteria were as follows: unstable situations during the assessment (recognized with the aid of the nurses), parents’ refusal of their children’s participating in the program despite their initial consent. 

Verbal informed consent to the children’s participation in the study was obtained from their parents, and the protocol design was approved by the Ethics Committee of Tehran University of Medical Sciences, Tehran, Iran. 

The instruments utilized in this study were 1) demographic questionnaire, which included the profile of an infant, sex, the birth weight, the gestational age, the date of the assessment, etc., and was filled using medical records, 2) Test of Infant Motor Performance (TIMP), and 3) Neonatal Behavioral Assessment Scale (NBAS). One of which was randomly selected and performed, and then, after a brief interval, the other tests were performed.

TIMP and NBAS measure the motor function and the behavioral function of infants, respectively. Both TIMP and NBAS tests are predictive tests. Among all infancy tests, TIMP is the strongest early infancy predictor test (15, 16). TIMP is utilized for the infants aged from 16 weeks to 32 weeks. It includes the observations of motions and elicited section for purpose of assessing the postural control and performance (1, 4, 10). NBAS is a test used for normal infants as well as the infants at risk aged from 36 weeks fetus to 2 months (1). It includes such items as habituation, social interactions, the motor system, the state organization, the state regulation, the autonomic system, smile, supplementary items, and the reflex.

TIMP has a very high validity and reliability (ICC=0.98) (4, 10, 15, 17). The validity of NBAS is high; however, its reliability turned out to be low to moderate. Nevertheless, because infancy is characterized by its rapid changes in the physical, physiological, and behavioral systems, throwing the reliability of this test into question is not prudent. Therefore, the low reliability of this test should not be considered as a deficiency of this test (9, 10). It just indicates its great sensitivity to changes.

For statistical analysis, we used SPSS version 20 (Chicago, IL, USA) and for investigation of between the independent variables with observational and elicited sections the linear regression model were used. In this model, the observation and elicited parts of TIMP were deemed as the dependent variable, and such variables as habituation, social interactions, the motor system, the state organization, the state regulation, the autonomic system, smile, supplementary items, and the reflex (all taken from NBAS test) were regarded as independent variables. Both dependent and independent variables were separately entered in the model, and the probable relationship between any of these independent variables with the dependent variable was gauged in terms of gender, age, and birth weight. The results were reported as β at 0.05 significance level. The relationship between variables was measured using the correlation coefficient. 

Since the variables in this study were ordinal, to calculate the correlation coefficients between variables, the Spearman correlation was used. Correlation coefficient exponentiation was also used to compute the determination coefficient (r2). The significance level in all tests was 0.05.

Sixty percent (n=30) and 40% (n=20) of the participants were male and female infants respectively. The birth type in 56% of them was natural, and 44% of them were born by Caesarean section. Fifty two percent of the participants were hospitalized in NICU, and 48% were ambulatory patients who went to the pediatric clinic. The results of the descriptive statistics are presented in Table 1. Table 2 provides the results of the statistical analysis. Accordingly, there was no significant correlation between the habituation of the behavioral test and the observation of the movement test (P: 0.77 and β: 0.06), nor between the habituation of the behavioral test and the motor section of the elicited test (P: 0.06 and β: 3.02). No significant relationship was found between autonomous systems and the observation section (P=0.06 and β=1.62). A significant correlation between the social interaction and the observation section was found since P =0.02 and β= 0.77, i.e. for each point of the social interaction score of the NBAS, 0.77 point was added to the score of the observation section of the TIMP. There was also a significant relationship between the social interaction (P=0.001 and β= 95.7) and the elicited items section, i.e., for each point of one score to the social interaction score, 7.95 points were added to the elicited items section score (Table 2).

**Table 1:** Descriptive Statistics of the Participants

**Variable**	**Standard deviation**	**Average**	**Maximum**	**Number**	**Minimum**
**Birth weight**	316.87	1992.200	2480	50	1500
**Term of hospitalization (day)**	7.17	7.62	35	50	1
**Birth age (week)**	3.36	36.26	42	50	30

**Table 2:** The Results of the Correlations between TIMP and NBAS Tests

		**TIMP**							
		**Observation**				**Elicited**			
		**β**	**R**	**R2**	**Level of significance β**	**β**	**R**	**R2**	**Level of significance β**
**NBAS**	**Habituation**	0.06	0.05	0.25	0.77	3.02	0.12	0.01	0.06
	**Social interaction**	0.77	0.36	0.12	0.02	7.95	0.37	0.13	0.001
	**Motor system**	1.02	0.58	0.33	0.00	8.41	0.64	0.40	0.00
	**State organization**	1.09	0.65	0.42	0.00	6.98	0.59	0.34	0.001
	**State regulation**	0.72	0.61	0.37	0.001	4.05	0.53	0.28	0.009
	**Autonomic system**	1.62	0.06	0.36	0.06	18.45	0.80	0.64	0.002
	**Smile**	1.47	0.36	0.12	0.02	13.19	0.45	0.20	0.004
	**supplementary items**	1.04	0.71	0.50	0.00	8.67	0.75	0.56	0.00
	**Reflex**	4.01	0.64	0.40	0.00	38.93	0.79	0.62	0.00

## Discussion

Although the NBAS has been used in several studies, no study has used this test to predict the motor function in infancy and babies less than two months age. In this study, in the linear regression, the observation and elicited sections were entered into the model as the dependent variables and the habituation of the NBAS as the independent variable. There was no statistically significant correlation between the habituation and observation items (P=0.77 and β=0.06). 

The results of this study were not in line with the findings of Ohgi et al. where the habituation as a behavioral item could be a good predictor of developmental problems (15). Habituation is defined as the conformity to the stimuli, gained more rapidly in some infants and postponed in others. Habituation is deemed as a self-protection skill against disturbing motions, which assists the infant to live with his family conveniently. Accordingly, the infants suffering from some problems with their internal systems are turbulent, so, are not able to habituate to stimulations. According to the section pertaining to the scoring instruction of the NBAS in the infants who cry early, the lowest score should be considered and the test should be ceased (10). In this study, whenever the infant cried during the TIMP, the infant was calmed down and the test was resumed. It is indicative of the great emphasis the NBAS placed on the stress-free condition of the infant.

A significant correlation between the observation section of the TIMP and the social interaction items of the NBAS was observed since β=0.77 and P= 0.02, in other words, for each point of the score of social interaction item, 0.77 point was added to the score of the observation section. 

There was also a significant relationship between the elicited item of the TIMP and the social interaction items of the NBAS (P=.001 and β= 7.95), i.e., for each point of the score of the social interaction, 7.95 points were added to the score of the elicited section. The correlation between the social interaction and any observation and elicited sections of the TIMP was also significant. Therefore, the social interaction score of the NBAS might be utilized to predict the infant’s motor function score. The results of the present study were consistent with those observed earlier (15, 18).

There was a significant correlation between the observation section of the TIMP and the motor system item of NBAS (P= 0.00 and β=1.02). It means that for each point of the score of the motor system item, 1.02 points were added to the observation score. A significant relationship between the elicited section of the TIMP and the motor of the NBAS (P=0.00 and β=8.41) was also achieved. It means for each point of the score of the motor item, 8.41 points were added to the score of the elicited item section; moreover, the correlation between these variables was also significant. Therefore, it can be deduced that it is feasible to utilize the NBAS scores to predict the TIMP scores. The NBAS motor test scores can be used as a strong predictor to envisage the later developmental disabilities (15, 18). A significant correlation was found between the observation part of the performance test and the organization item (P= 0.00 and β= 1.09), meaning that, for each1 point increase in the score of the organization item, 1.09 points were added to the observation score. Similarly, there was a correlation between the elicited section of the TIMP and the state organization item of the NBAS (P=. 001 and β= 6.98) for each point of the score of the state organization item, 6.98 score was added to the elicited score. The correlation between these variables was also significant. 

Therefore, perhaps the NBAS cases can be predicted by the item scores for the physical exam. The results of the present study are in line with those reported earlier (15, 18).

A significant correlation was observed between the observation section of the TIMP and the smile item in the behavioral test (P=0.02 and β= 1.47), in other words, for each point of the score of the smile item, 1.47 points were added to the score of the observation part. 

A significant relationship between the elicited section of the TIMP and the elicited item was found, i.e., for each point of the score of the smile item, 4.05 points were added to the stimulation score. The correlation coefficient between these variables was also statistically significant. Thus, it might be feasible to utilize the score of the smile item in the NBAS to predict the infant’s TIMP scores. A significant correlation was observed between the observation section of the TIMP and the supplementary item (P=0.00 and β=1.04); to put it in other terms, for each point of the supplementary scores, 1.04 points were added to the observation scores. 

Besides, a significant correlation was found between the elicited section and the supplementary item of the NBAS (P=0.000 and β=8.67), i.e., for each point of the supplementary score, 8.67 points were added to the elicited score; and the correlation between these variables was significant.

There was a significant correlation between the observation section of the TIMP and the reflex item of the NBAS (P= 0.00 and β= 4.01), i.e., for each point of the reflex score, 4.01 points were added to the score of the observation section. Moreover, a significant relationship was found between the elicited section of the TIMP and the reflexes item of the NBAS (P=0.000 and β=38.93), in other words, for each point of the reflex score, 38.93 points were added to the elicited score; and the correlation between these variables was also significant. 

It might be possible to use the social reflex item scores of the NBAS score to predict the infant’s motor function. 

The results of the present study are not aligned with those of Ohgi et al.’s study (15). They concluded that NBAS could be served as a good predicator of later developmental disabilities, and the high scores obtained from the reflex item could be a strong predictor of future developmental disabilities (15). 

No significant relationship was seen between the autonomous system and the observation section (P=0.06and β=1.62); consequently, the autonomous item score cannot be used to predict the score of the observation section of the TIMP. There was a significant correlation between the elicited section of the TIMP and the autonomous system of the NBAS (P=0.002 and β=18.45), i.e., for each point of the score of the autonomous system item, 18.45 points were added to the provocation score. Therefore, probably, the autonomous system item score can be used to predict the score of the elicited section of the TIMP.

**In conclusion, **TIMP is regarded as the strongest predictor among all other newborn tests; however, this test imposes some stress on infants. It might be proclaimed that via using the scores of most items of the NBAS, TIMP can be determined.
